# He hurts her womb: Physical-sexual violence and pregnancy complications among women in Afghanistan

**DOI:** 10.34172/hpp.2021.61

**Published:** 2021-12-19

**Authors:** Mostafizur Rahman, Priom Saha, Nahida Anwar, Afnan Hossain

**Affiliations:** ^1^Department of Science and Humanities, Bangabandhu Sheikh Mujibur Rahman Aviation and Aerospace University, Old Airport, Tejgaon, Dhaka 1215, Bangladesh; ^2^Institute of Statistical Research and Training, University of Dhaka, Dhaka 1000, Bangladesh; ^3^Department of Philosophy, University of Dhaka, Dhaka 1000, Bangladesh; ^4^Department of Peace and Conflict Studies, University of Dhaka, Dhaka 1000, Bangladesh

**Keywords:** Intimate partner violence, Physical abuse, Pregnancy complications, Sex offenses, Women

## Abstract

**Background:** Though some studies show the association between intimate partner violence and pregnancy complications in developed countries, the association remains understudied in less developed and low-income settings. This study examines the association of physical and sexual violence with pregnancy complications among women in Afghanistan.

**Methods:** This study used the data from the 2015 Afghanistan Demographic and Health Survey (AfDHS). The analysis included 7229 women aged between 15 and 49 and used logistic regression to show the association of physical and sexual violence with pregnancy compilations. The analysis controlled for some potential variables and followed complex survey design factors such as strata, clusters, and survey weights.

**Results:** Fully adjusted regression model shows that the women who experienced physical violence were 21% (adjusted odds ratio [OR]=1.21; confidence interval [CI]=0.98, 1.50; *P* <0.1) more likely to endure pregnancy complications compared to those who did not face the violence. Likewise, the women experiencing sexual violence were 89% (adjusted OR=1.89; CI=1.37, 2.62; *P* <0.01) higher to face pregnancy complications than those who did not face any of sexual violence. More specifically, physically and sexually violated women were highly prey to the complications that increased with the increment of the violence.

**Conclusion:** This study adds that policymakers may formulate policies for ensuring 3C (caring couple counselling) by readdressing couple relationships, raising gender rights and awareness, providing reproductive health literacy, and increasing mental health awareness during pregnancy.

## Introduction

Pregnancy complications are the leading cause of maternal mortality all over the world. Despite substantial progress made in reducing maternal mortality over the past decades, every day approximately 810 women die worldwide due to pregnancy and childbirth-related complications. About 295 000 women died globally in 2017 from complications during pregnancy or childbirth and 94% of these deaths occurred in low to middle-income countries.^1^ Since the pregnancy rate is higher among women in less developed countries, their lifetime risk of death due to pregnancy complications is more severe. For instance, the maternal mortality ratio (MMR) is 462 per 100 000 live births in low-income countries compared to, 11 per 100 000 live births in high-income countries.^1,2^ Complications occurred during pregnancy mostly include severe bleeding, infections, high blood pressure (eclampsia), delivery difficulties, and unsafe abortions which are accounted for nearly 75% of all maternal deaths.^1,3,4^

Pregnancy complications are the results of various factors including poor and/or limited access to prenatal care, lack of nutrition, previous history of illness (i.e., diabetes, cancer, high blood pressure, sexually transmitted disease, kidney problems, epilepsy, anemia, infection), and drug abuse.^5-9^ Another major cause of pregnancy complications is physical and sexual violence against women. Several previous studies have evidenced that physically and sexually abused women are more likely to experience the complications.^4,10-12^ Particularly, physical and sexual violence during the pregnancy period may increase the risk of miscarriage,^13^ premature delivery,^14^ low gestational and birth weight,^15,16^ placental abruption,^17^ poor fetal growth, and injury-related death.^18^

Violated women experience adverse psychological outcomes^19,20^ that cause severe perinatal maternal stress and trauma which result in dysregulation of neuroendocrine, neural, immune, and maternal hypothalamic-pituitary-adrenal (HPA) axis system.^21-24^ The dysregulation of the HPA axis determines the exposure of stress hormones such as cortisol^23^ that limit the blood flow to the uterus, and constrict the soft muscle tissue of the uterus, and cause preterm labor, premature birth, and low birth weight.^25,26^ The World Health Organization (WHO) reported that women who experience physical-sexual abuse during pregnancy are almost two times more likely to experience depression compared to women who have not experienced partner violence.^2^

Globally, 35% of women experience some form of physical and sexual violence during their lifetime. The prevalence of violence is more severe in underdeveloped and developing countries.^2^ Afghanistan as a least developed country has a higher rate of physical-sexual violence against women. For instance, the 2015 Afghanistan Demographic and Health Survey (AfDHS) depicts that about 46% of the women in Afghanistan experience some sort of physical-sexual violence.^27^ Eventually, Afghanistan has one of the highest MMRs in the world (638 per 100 000 live births).^1^ An estimated 40%-50% of deaths of reproductive-aged women in Afghanistan are related to pregnancy complications and childbirth. According to the Afghanistan Mortality Survey 2010, about 3-5 women die during pregnancy or childbirth or two months after delivery for every 1000 live births.^28^ Studies evidence that despite women’s unwillingness, they feel obligated to engage themselves in sexual activities and later neglect reporting the physical and sexual violence because of religious and cultural motivations.^29^ The unreported cases may increase or add to the present prevalence rate of violence and adverse consequences.

The existing literature divulges that the association of physical-sexual violence with pregnancy complications is examined in some developed countries, but the association is understudied in low-income settings like Afghanistan. Hence, this study aims to address the gap by examining the association of physical-sexual violence with pregnancy complications, using the 2015 AfDHS.

## Materials and Methods

### Data

De-identified data from the 2015 AfDHS were used in this study. Like other Demographic and Health Surveys (DHS) conducted in almost 90 countries, AfDHS is a nationally representative household survey and collects demographic and health-related information including fertility, mortality, nutrition, immunization, healthcare utilization, and other queries from women aged between 15 and 49. In 2015 AfDHS, 25 741 households were chosen for the survey and 24 395 of them were interviewed between June 15, 2015, and February 23, 2016. Each of the respondents was asked for providing informed consent. The collected data were entered twice for verification and accuracy. The response rate of the survey was 98%. After the exclusion of the observations with missing values, our analysis used a sample of 7229 reproductive-aged women (15 to 49).

### Outcome variables

The outcome variable of this study is the pregnancy complications of reproductive-aged women. AfDHS asked each married woman, whether the doctor “told about pregnancy complications at the time of last antenatal visit”. The ‘yes’ response was given 1 and the ‘no’ response was given 0, while ‘don’t know’ was put as missing.

### Key explanatory variables

*Physical violence:* Women’s lifetime experience of any physical violence was one of the key exposure variables in this analysis. Women were asked about their lifetime experience of physical violence with 7 queries: “whether her husband ever pushed, shook or had something threw at her; slapped her; punched her with his fist or with something that could hurt her; kicked her, dragged her or beat her up; tried to choke her or burn her on purpose; threatened or attacked her with a knife, gun, or any other weapon; or twisted her arm or pulled her hair.” The responses were ‘never’, ‘often’, ‘sometimes’, ‘yes, but not in the last 12 months’, and ‘yes, but frequency in last 12 months missing’. We coded ‘never’ 0 and coded the rest 1 and thus created the lifetime experience of women’s physical violence. Total score varied from 0 to 7, where 0 meant no violence and the rest (1-7) indicated women’s lifetime exposure to physical violence.

*Sexual violence:* Another key exposure variable was sexual violence of women that was measured with three variables. In AfDHS, women were asked,“whether her husband ever physically forced her to have sexual intercourse with him even when she did not want to; or forced her to perform any sexual acts; or forced into other unwanted sexual acts”. The response items were the same as those of physical violence variables such as ‘never’, ‘often’, ‘sometimes’, ‘yes, but not in the last 12 months’, and ‘yes, but frequency in last 12 months missing’. Like the physical violence measure, ‘never’ was coded 0 and the rest was coded 1 and in the same way, a lifetime exposure to sexual violence was created based on these three items.

*Wealth index:* The wealth index is a composite measure that is calculated based on selected household assets, and dwelling characteristics including water access, sanitation facilities, materials used for house construction, bicycles, televisions, and others.^30^ Individual households are given a continuous score which is, using principal component analysis, then categorized into five wealth quintiles; poorest, poorer, middle, richer, and richest. Wealth is applied in health and demographic studies to examine the effects of wealth on maternal and child health outcomes such as nutrition, family planning, and others.^31^ In this study, the poorest and poorer categories as well as the richest and richer groups were merged and were made poor and rich categories, respectively.

*Exposure to mass media index:* Each woman replied to three queries about their frequency of exposure to three mass media; (a) television, (b) radio, and (c) newspaper. In this index, ‘not at all’ was coded 0, while ‘less than once a week’, ‘at least once a week’, and ‘almost every day’ were coded 1. The total summed score ranged between 0 and 3. The women who scored 0 were considered as having no exposure to mass media and those who scored 1-3 were indicated as having exposure to mass media.

*Wife-beating justification index:* Beating justification of wife is a measure that was summed up with five items regarding their perception about the violence committed by their partners/husbands against them: (a) “Beating justified if wife goes out without telling husband”, (b) “Beating justified if wife neglects the children”, (c) “Beating justified if wife argues with husband”, (d) “Beating justified if wife refuses to have sex with husband”, and (e) “Beating justified if wife burns the food”. For the responses of each query, the ‘no’ response was coded 0, the ‘yes’ response was coded 1, and ‘don’t know’ was coded missing. Later, all items were counted together and the total count was scored between 0 and 5, where score 0 meant beating as not justified and score 1-5 indicated beating as justified among women.

*Covariates:* The analysis adjusted for some potential factors. Those included respondents’ current age (in years), place of residence (rural, urban), total number of children, age at marriage (<18, ≥18), education (in years), occupational status (not occupied, occupied), wealth status (poor, middle, rich), antenatal care (ANC) visits (0-3, ≥4), and type of contraceptive use (no method, traditional, modern).

### Statistical analysis

First, descriptive statistics were used to present sample characteristics. Then, the association of physical and sexual violence with pregnancy complications was assessed using logistic regression analysis, where we fitted three models and adjusted for some potential variables. Model 1 adjusted for socio-demographic factors (respondent’s current age, place of residence, total number of children, age at marriage, and education in years), model 2 further adjusted for economic factors (occupational and wealth status), and model 3 further adjusted for mass media exposure, justification of wife-beating, ANC visits, and type of contraceptive use. The regression analysis followed complex survey design factors such as strata, clusters, and survey weights. The confidence interval (CI) was set at 95% and a *P* value less than 0.1 or 0.05 or 0.01 was considered significant. STATA 14 (StataCorp LP, College Station, TX) was used for all analyses.

## Results

Table 1 presents the socioeconomic, demographic, and health behavioral characteristics of women by physical and sexual violence. Women of physical and sexual violence have 29 years of mean age with a standard deviation of nearly 7 years. The result shows that the women who are exposed to physical violence have lower education (1.13 mean years), whereas the women who are not exposed to physical violence have comparatively higher education (2.22 mean years). Similarly, women with exposure to sexual violence have less than a year (0.98) of education that is lower than those who did not have sexual violence (1.76 mean years). About three-quarter of women who are exposed to physical violence (76%; CI = 72%, 80%) and sexual violence (74%; CI = 72%, 82%) are rural residents. Women who have no occupation are more prone to physical and sexual violence. For instance, 87% (CI = 82%, 90%) of the women exposed to physical violence and 85% (CI = 80%, 90%) of the women exposed to sexual violence are not occupied. Women of poor (38% physical violence; CI = 33%, 43% and 45% sexual violence; CI = 37%, 52%) and rich categories (42% physical violence; CI = 37%, 47% and 41% sexual violence; CI = 33%, 51%) faced more violence compared to the middle (21% physical violence; CI = 18%, 23% and 14% sexual violence; CI = 10%, 19%). Women aged under 18 years were more victim of physical (52%; CI = 50%, 55%) and sexual violence (54%; CI = 47%, 60%) and women with more number of children (4.60 for physical and 4.43 for sexual) tolerated more violence.

**Table 1:** Sample characteristics of women by physical and sexual violence (N = 7229)

**Variables**	**Physical violence**			**Sexual violence**		
	**No** **% (N)**	**Yes** **% (N)**	**Total** **% (N)**	**No** **% (N)**	**Yes** **% (N)**	**Total** **% (N)**
Current age*	29.03 (6.85)	29.85 (6.92)	29.42 (6.90)	29.18 (6.94)	29.17 (6.40)	29.42 (6.90)
Education (years)*	2.22 (4.15)	1.13 (2.95)	1.70 (3.67)	1.76 (3.73)	0.98 (2.75)	1.70 (3.67)
Place of residence						
Rural	0.66 (2247)	0.76 (2910)	0.71 (5157)	0.71 (4805)	0.74 (351)	0.71 (5157)
Urban	0.34 (1169)	0.24 (902)	0.27 (2072)	0.29 (1949)	0.26 (123)	0.29 (2072)
Occupation						
Not occupied	0.87 (2982)	0.87 (3307)	0.87 (6289)	0.87 (5884)	0.85 (405)	0.87 (6289)
Occupied	0.13 (434)	0.13 (506)	0.13 (939)	0.13 (870)	0.15 (69)	0.13 (939)
Wealth Index						
Poor	0.32 (1092)	0.38 (1433)	0.35 (2526)	0.34 (2315)	0.45 (211)	0.35 (2526)
Middle	0.18 (607)	0.21 (792)	0.19 (1398)	0.20 (1332)	0.14 (67)	0.19 (1398)
Rich	0.50 (1717)	0.42 (1588)	0.46 (3304)	0.46 (3108)	0.41 (197)	0.46 (3304)
Age at marriage						
<18 years	0.52 (1777)	0.52 (1999)	0.52 (3776)	0.52 (3521)	0.54 (255)	0.52 (3776)
≥18 years	0.48 (1639)	0.48 (1813)	0.48 (3452)	0.48 (3233)	0.46 (220)	0.48 (3452)
Number of children*	4.27 (2.66)	4.60 (2.63)	4.43 (2.65)	4.43 (2.66)	4.43 (2.44)	4.43 (2.65)
Contraception use						
No method	0.71 (2428)	0.62 (2356)	0.66 (4783)	0.67 (4454)	0.70 (330)	0.66 (4783)
Traditional	0.05 (186)	0.04 (158)	0.05 (343)	0.05 (321)	0.05 (22)	0.05 (343)
Modern	0.24 (803)	0.34 (1299)	0.29 (2102)	0.29 (1979)	0.26 (122)	0.29 (2102)
ANC visit						
0-3 visits	0.65 (2172)	0.70 (2644)	0.68 (4817)	0.67 (4490)	0.70 (326)	0.68 (4817)
≥4 visits	0.35 (1185)	0.30 (1128)	0.32 (2313)	0.33 (2172)	0.30 (141)	0.32 (2313)
Mass Media Exposure						
Not exposed	0.26 (898)	0.26 (988)	0.26 (1886)	0.26 (1756)	0.27 (130)	0.26 (1886)
Exposed	0.74 (2518)	0.74 (2824)	0.74 (5342)	0.74 (4998)	0.73 (345)	0.74 (5342)
Beating Justified						
Not justified	0.25 (852)	0.10 (393)	0.17 (1244)	0.18 (1195)	0.10 (49)	0.17 (1244)
Justified	0.75 (2564)	0.90 (3420)	0.83 (5984)	0.82 (5559)	0.90 (425)	0.83 (5984)
Total	3416	3813	7229	6754	475	7229

*Mean (SD)

Among the women exposed to sexual violence, 70% used no contraceptive methods, while only 26% of them used modern contraceptives. Likewise, 62% of women experiencing physical violence used no contraceptive method, whereas only 34% of them used modern contraceptives. We found, 70% of the women exposed to physical and sexual violence received less than 4 ANC visits, leaving only 30% with 4 or more ANC visits. In addition, 90% of the women exposed to physical and sexual violence assumed that husbands were justified in beating their wives.

Table 2 shows the bivariate association of physical and sexual violence with pregnancy complications. Here, physical and sexual violence are highly associated with pregnancy complications at 5% level of significance. 64% of the women who were exposed to physical violence have pregnancy complications and 74% of the women who experienced sexual violence have pregnancy complications.

**Table 2:** Bivariate association of physical and sexual violence with pregnancy complications (N = 7229)

**Variables**	**Pregnancy Complications**		**χ** ^ 2 ^ ** (** ***P*** ** value)**
	**No** **% (N)**	**Yes** **% (N)**	
Physical violence			
No	0.42 (1419)	0.58 (1997)	23.01 (0.02)
Yes	0.36 (1365)	0.64 (2448)	
Sexual violence			
No	0.39 (2662)	0.61 (4092)	31.78 (<0.001)
Yes	0.26 (122)	0.74 (352)	

Table 3 exhibits the adjusted odds ratios (ORs) and 95% CIs of physical and sexual violence. In model 1, adjusting for the socio-demographic variables, the odds of pregnancy complications is 28% (OR = 1.28; CI = 1.04, 1.57; *P* < 0.05) higher for the women exposed to physical violence compared to those who were not exposed. The odds of pregnancy complications are 92% (OR = 1.92; CI = 1.41, 2.60; *P*< 0.01) higher for the women who were exposed to sexual violence than those who were not exposed. Further adjusting for occupational and wealth status in model 2, a similar result was found for physical violence, but the OR attenuated a bit for sexual violence (OR = 1.89; CI = 1.39, 2.57; *P* < 0.01). In model 3 (further adjusting for mass media exposure, justification of beating, ANC visits, and type of contraceptive use), the odds of pregnancy complications for the women exposed to physical violence attenuated a bit (OR = 1.21; CI = 0.98, 1.50; *P* < 0.1), but the OR of sexual violence for pregnancy complications remained the same as the model 2 (OR = 1.89; CI = 1.37, 2.62; *P* < 0.01). The findings imply that the women who experienced physical and sexual violence were prey to pregnancy complications.

**Table 3:** Adjusted odds ratios and 95% confidence intervals for physical and sexual violence (N = 7229)

**Variables**	**Logistic regression model of pregnancy complications**		
	**Model 1**	**Model 2**	**Model 3**
	**OR (95% CI)**	**OR (95% CI)**	**OR (95% CI)**
Physical violence	1.28** (1.04, 1.57)	1.28** (1.04, 1.57)	1.21* (0.98, 1.50)
Sexual violence	1.92*** (1.41, 2.60)	1.89*** (1.39, 2.57)	1.89*** (1.37, 2.62)

*** *P* < 0.01, ** *P* <0.05, * *P* <0.1 Model 1: Adjusts for respondent’s current age, place of residence, total number of children, age at marriage, and education in years. Model 2: Further adjusts for occupational status, and wealth status. Model 3: Further adjusts for mass media exposure, justification of beating, antenatal care (ANC) visits, and type of contraceptive use.

## Discussion

Pregnancy complications have been major challenges and threats to maternal and neonatal health. Therefore, pregnancy complications have been important and prior concerns in plan and policy initiatives, for instance, Sustainable Development Goal 3 that ensures good health and wellbeing for all irrespective of gender and age. Hence, controlling the associated factors has been a primary concern to reduce the complications and to improve maternal and neonatal health. In this motive, this study examined the association of physical and sexual violence with pregnancy complications in Afghanistan.

The findings of this study strongly evidenced that the women who experienced lifetime physical and sexual violence were at higher risk of pregnancy complications. The analysis reports that about two-third of pregnancy complications were experienced by the women who were the victims of physical violence. Also, about three a quarter of pregnancy complications were endured by the women who were exposed to sexual violence than those unexposed. Similarly, a study found that about four in every five physically and sexually abused women faced pregnancy complications.^32^ Other studies also found the same association between violence and unwanted reproductive outcomes.^33-35^ We hypothesized from the precedent studies that physical and sexual violence cause pregnancy complications through different physiological and psychological mechanisms. This study to some extent discusses psychological mechanisms through which physical and sexual violence impact maternal physical reproductive health. Thus, the results further confirm that physical and sexual violence are threats to maternal reproductive as well as child health. Hereby, we suggest that policymakers may formulate policies for ensuring 3C (caring couple counselling) by readdressing couple relationships, raising gender rights and awareness, providing reproductive health literacy, and increasing mental health awareness during pregnancy.

The findings demonstrated that physical violence has a robust association with pregnancy complications. More specifically, the association suggests that pregnancy complications were higher among women who were physically coerced compared to those who were not coerced. Similarly, previous studies reported the same association between physical violence and pregnancy complications.^17,32,36,37^ The reason behind the association is that hurting a woman physically may directly cause harm to physical maternal and fetal health,^36,38-40^ mental conditions^19,20,41,42^ and hormonal imbalance as well.^43,44^ Even, the violated women have a less protective mechanism, as they are less possible to get emotional support, ANC visits, medical advice, and healthcare facilities.^45,46^

This study divulged that sexual violence has evolved as a strong predictor of pregnancy complications. More particularly, sexual violence increased the likelihood of pregnancy complications that were pronounced among women who faced the violence (sexual) compared to those who did not face that. Prior studies, similar to our findings, showed pregnancy complications have been echoed among women who faced sexual violence.^32,33,47^ It happens because pregnancy complications occur due to either physical harm or hormonal imbalance, both of which are associated with perinatal stress. Living with an abusive partner is exceedingly stressful and thus injurious to maternal reproductive health.^10,48^ Clinical studies evidence that stress during pregnancy increases cortisol exposure that contracts blood vessels and restricts blood flow to the uterus and in this way causes pregnancy complications.^49,50^

The social hierarchy of masculinity and femininity and gender norms and roles in low- and middle-income countries (LMICs) are significant factors of intimate partner and sexual violence.^51^ The possibility of sexual violence is related to the degree to which the belief in masculinity and the enjoyment of sexual rights by men is deeply embedded (in the community). Disagreements often occur over traditional gender roles, differences in social-economic status, and sexual behavior or rejection of control in partners.^51^ Evidence shows that this continuous disagreement often results in low marital satisfaction and violence is often used as a way of dealing with the conflicts or resolving the differences.^52-54^ However, study shows that these forms of violence can be resolved through counselling like systematic couples therapy and relationship enhancement education and counselling and thus recommends reducing physical, psychological and sexual violence against women during pregnancy in LMIC settings.^55^

The first key strength of this study is its use of a nationally-representative survey and it can thus be generalized at the national level. Second, the pregnancy complications provide an accurate estimate, as it was reported through medical procedural reports by the trained physician during ANC visits. Despite these strengths, we acknowledge some limitations as well. As it is a cross-sectional analysis, it does not allow to infer causal interpretations. Some variables were controlled in the analysis (respondent’s current age, place of residence, number of children, age at marriage, education, occupation, wealth status, mass media exposure, justification of beating, ANC visits, and method of contraceptives). However, we were unable to control some other factors such as hemoglobin level, maternal height and weight, lineage characteristics, intention of pregnancy, adverse pregnancy history, access to health care centers, household environment, and husband’s socio-economic conditions. We suggest that future studies can be performed by considering these shortcomings.

## Conclusion

As the leading causes of maternal and child mortality, pregnancy complications have currently been considered as major public health concerns and development challenges. Current study divulged that women who faced intimate partner violence physically and sexually were at higher risk of enduring pregnancy complications compared to those who did not face the violence. In other words, the increment in physical-sexual violence increases pregnancy complications among ever-married and reproductive-aged women. Continual living with an abusive partner is stressful and it causes pathophysiological symptoms that constrict fetal growth and causes harm to maternal health. Therefore, abating physical and sexual violence through policy interventions may be effective to fight against prenatal and postnatal complications of women. In this regard, current study suggests, the policymakers may emphasize 3C (caring couple counselling) to increase couple relationships, gender rights and awareness, reproductive health literacy, and mental health awareness during pregnancy.

## Funding

The authors received no funding for this study.

## Competing interests

None.

## Ethical approval

Not required.

## Authors’ contributions

MR designed the study. PS analyzed the data and interpreted the results. AH drafted the introduction and corrected the references. MR and NA wrote the methods, discussion, and conclusion.

## Data sharing

Data used in this study is publicly available on the following web page of the DHS Program.

(https://dhsprogram.com/data/available-datasets.cfm).
